# The evolution of barriers and facilitators to using a COPD app among older adults: results from a pilot study

**DOI:** 10.3389/fdgth.2025.1557590

**Published:** 2025-07-14

**Authors:** Jeff Brady, Rachel F. McCloud, Erin Higgins, Aishwarya Mahesh, Keith LeJeune, Jon Black, Anil Singh

**Affiliations:** ^1^ Highmark Health, Pittsburgh, PA, United States; ^2^ JSI Research & Training Institute (United States), Atlanta, GA, United States

**Keywords:** mHealth, older adults, chronic disease, COPD, technology

## Abstract

**Purpose:**

Although older adults are more frequently adopting smartphone technologies, factors influencing decisions for uptake and continued use remain complex. This study explored how perceptions and use of a smartphone app changed from pre-adoption through initial use.

**Methods:**

Participants were interviewed before, during, and after being introduced to a COPD app to assess their experiences with and perceptions of the app over a 4-month period.

**Results:**

Prior to app introduction, participants reported technology, health behavior, and contextual barriers to engaging with health technology. After app introduction, many technology-based barriers lessened over time as participants became more familiar with the app. Other barriers, such as perceived lack of relevance and competing health and life concerns, remained as challenges to use.

**Discussion:**

Results point to the need for apps that can cater to the diverse needs and other life challenges of older adults. Opportunities for assistance from technical support lines, family, friends, and/or community are required.

## Introduction

Over the past decade, there has been a proliferation of health-related smartphone applications (apps) available for consumers (1, 2). Mobile health, or mHealth, offers the promise of delivering health information and social connection for myriad health conditions across the lifespan (3). Health apps, when effective, could be beneficial for older adults as they have the highest rates of chronic disease compared to any other age group (4), and have a high risk of mental health concerns (5). However, research indicates more action should be taken to ensure the appropriate fit of specific apps—and their available technical support–with their target populations (6–8).

When it comes to health apps, one size does not fit all, particularly for older adults. Historically, adults age 65 and older have been identified as the group with the lowest use of new technologies, with those who are older, lower income, and have a lower level of education less likely to use health apps than their counterparts (9). Lack of access to devices and internet connections have been cited as major barriers to technology use, as well as low digital self-efficacy, low awareness of app offerings, distrust of technology, legal concern, and lack of technology-based skills and support systems (10–13).

Technology-based factors associated with health apps are intertwined with an individual's health-related knowledge, attitudes, and behaviors, which are also impacted by a complex interplay of individual, interpersonal, and societal factors (13–16). mHealth users must use a range of digital skills to navigate through an app to gain information, complete tasks, and apply learning within different life contexts (17). Completing this process may be challenged by multiple comorbidities, in which complex care needs for several conditions may result in competing priorities that require strict scheduling or more mental capacity (18). Furthermore, older adults must have trust not only in technology itself, but perceive that the doctors providing information and services on the mhealth application are credible as well (13).

However, focusing explicitly on the reasons why technology is *less* used by older adults risks perpetuating the stereotype that all older adults are technology avoidant (19). It is also important to acknowledge the *facilitators* to technology use by this population to identify ways to leverage these factors to increase use. Adoption of online technologies including tablets and smartphones by adults aged 65 and older has grown markedly in the past decade (20). Since the onset of the COVID-19 pandemic, older adults are online more than ever before, using the internet to seek out connection and address their health (21). Once connected to technology, many older adults often have robust online lives, using diverse technology features to play games, maintain social connections, and listen to music (22). Even among those who had initial hesitancy to use technology, many older adults can quickly adapt and begin multi-faceted use once access barriers are removed (23, 24).

The Unified Theory of Acceptance and Use of Technology (UTAUT) presents a synthesis of models to explain user acceptance and use of technology (24). In the UTAUT, factors including performance expectancy, effort expectancy, social influence, and facilitating conditions, along with moderators (i.e., age, sex), influence the behavioral intention to use technology, which in turn leads to actual technology use. For example, older adults assess the potential for usefulness and enjoyment that the technology could bring in their everyday life (performance expectancy) when determining if they will begin or continue use (25). Social motivation, encouragement, and support from family and friends are also an important facilitators for use (21).

Additionally, the importance and influence of certain barriers and facilitators may ebb and flow across the journey from non-use, to initial adoption, to sustained engagement (13). The use of the UTAUT to address multiple time periods (i.e., user adoption, initial use, and post-adoptive use) (26, 27) suggests that there may be opportunities to use this theory as a guide to identify and address critical, potentially modifiable issues at certain time points to increase the likelihood of uptake of a certain app. This may be particularly relevant for apps addressing health conditions, as continued use of disease management apps is suggested to gradually lead to acceptance after a period of use (28). Providing individuals with a specific health application will allow for observation of what technology- and health-based barriers persist or fade before and during an initial trial period.

**Figure 1:**
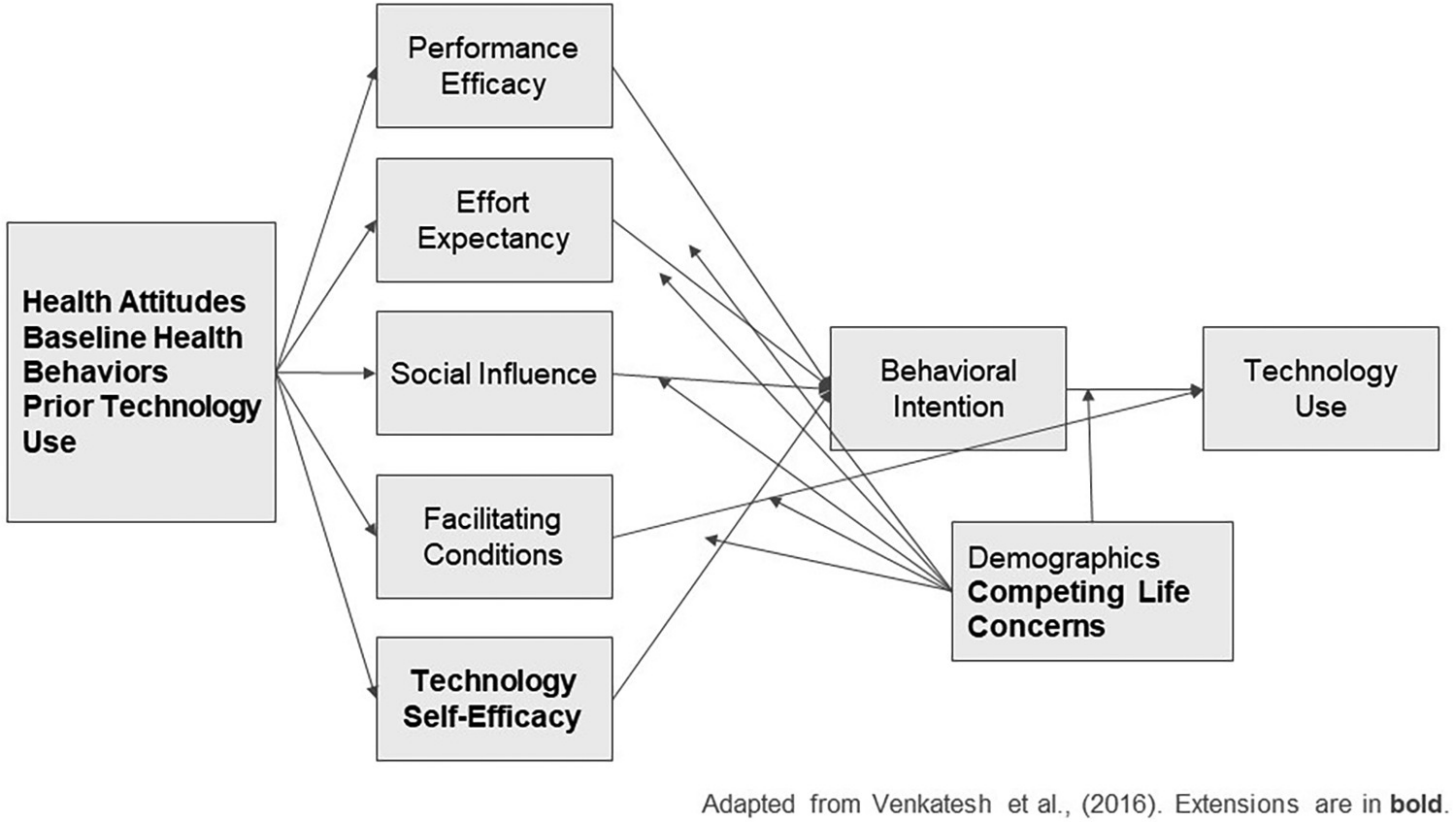
Adapted version of the unified theory of acceptance and use of technology.

Considering potential health-related barriers and facilitators as potential considerations in addition to established UTAUT constructs may be useful for understanding the initial adoption and ongoing use of health-related content. For example, Yu and Chen (29) found that a version of the UTAUT extended by factors related to perceived health served as a useful tool for exploring older adults' intention to use chatbots. Figure 1 depicts the UTAUT with additional factors (illustrated in bold) that may impact health app use. When introduced to new potential technology, individuals bring their previously held attitudes towards health (e.g., their perceptions about the severity of their health conditions, or their interest in improving their health) and health behaviors (e.g., steps they have or currently are taking to address their health) to the table when forming opinions about the attributes of the technology that may influence intention and use. In addition to challenges regarding health-related characteristics, apps addressing chronic conditions such as chronic obstructive pulmonary disease (COPD) often may face challenges during introduction of the technology based on patients' digital literacy or perceived technology self-efficacy, or barriers to ongoing use from competing life concerns or demands (13, 28–30). Taken together, this suggests a complex picture of both health and technology-related factors that may ultimately shape the decision to adopt and use a smartphone app.

The purpose of this study was to conduct an initial pilot study, guided by the UTAUT, of the introduction of a smartphone app to older adults who have both low reported technology engagement and COPD. A qualitative approach was selected for this study given the wide range of potential factors that might influence perceptions at any particular time point. Participants were interviewed before, during, and after app introduction to assess the experiences and perceptions of older adults as they move from low- or non-engagement with health technology through the initial adoption phase of a health-related app for COPD.

## Methods

The project was led by a large health care organization with operations that include health insurance, health care delivery, and other core activities. This qualitative study used interviews at several time points (prior to use, at initial app introduction, and the first four weeks after adoption) to capture the ongoing reactions of older adults as they began to engage with an app for COPD to learn how to maximize current and future health app offerings for their membership base. The technology application used for the study was designed to be used by patients to provide behavior change support and health information, including chats with health coaches, symptom tracking, and social forums. In addition to specific information about COPD, the app also provides assistance for other physical and behavioral health conditions. Participants used a study version of the app so many of the features, including video visits with health coaches and consults with physicians, were not operable. The Highmark Health IRB approved this research study.

### Sample/recruitment

Inclusion criteria were individuals who were 65 or older, self-identified as having COPD, reside in Southwestern Pennsylvania (PA), and a member of a Medicare Advantage Plan, a plan that provides healthcare services through private insurers that often includes extra benefits and care coordination. Eligible participants were also required to indicate a low level of engagement with their health and tech use, including not taking steps to improve their health and wellness, and infrequent to no use of health-related technologies. Finally, individuals had to agree to an initial in-home ethnographic interview and a 4-week trial simulating use of the app.

Recruitment and data collection were performed by a market research firm located in Pittsburgh, PA. Project personnel selected individuals from the firm's participant lists that met the eligibility criteria and sent them an email invitation to join the study. Interested individuals who responded to the email were then screened for participation.

In Phase 1, a trained researcher conducted a recorded, in-person 90-min formative interview with each participant. Semi-structured interviews included questions about participants' technology perceptions, barriers to technology use, health conditions, and barriers to healthy behaviors.

In Phase 2, participants were given a tablet preloaded with an app focusing on COPD care. Participants were instructed to sign into a test version of the app and engage with the content to explore different tasks and topic areas. Participants received: (1) a 90-min Zoom-based interview between weeks 1 and 2 that focused on initial impressions of the app and any potential challenges faced; (2) a series of brief weekly check-in calls to ask about ongoing perceptions of app utility or challenges; and (3) a 30-min exit interview at the end of the study to assess final impressions of the app.

### Measures

Semi-structured interview content and observation prompts were guided by UTAUT constructs and included other potential barriers to technology use identified through literature review and areas of interest identified by the organization. Effort expectancy was measured in Phase 1 through a series of questions asking participants to recall past technology engagement and to describe difficulties experienced during use. They were then asked to discuss any challenges they anticipated encountering when using technology in the future. In Phase 2, interviewers asked participants to recall their first impressions of the app at unboxing, including anticipated difficulties when reading the instructions and opening the app for the first time. Researchers then asked participants about their experiences when using the app and if anticipated difficulties arose. Performance expectancy was measured in Phase 1 through asking about general impressions of mhealth apps and what characteristics they used to determine if the technology was beneficial or valuable. In Phase 2, researchers asked participants about what COPD app features and other health topic content they perceived to be beneficial for their own health. Regarding social influence, participants were asked what role encouragement from family, friends, and health professionals played in past technology use. Facilitating conditions were assessed by asking participants about assistance they had sought using technology in the past and their interactions with and perceptions of the customer support services available for the study app. Prior technology use was explored in Phase 1, with a series of questions asking about the extent of past technology use on computers and smartphones. We also included questions about our proposed UTAUT extension, technology self-efficacy, through asking participants about how confident they felt about using technology and health apps in the past, and then asking about confidence in using the study app specifically during unboxing and as they begin to engage with the current app. In Phase 1 and 2, researchers asked participants about health-related factors related to COPD (e.g., perceived disease severity and how much it impacted their daily life). Phase 1 also explored barriers to health behaviors such as disease management and general health maintenance (e.g., smoking, healthy behaviors), asking about motivation to perform these behaviors as well as any relevant knowledge and health beliefs. The presence of any current competing concerns such as caregiving, other health conditions, or other challenging life circumstances, was also noted within all information gathering time points.

### Analysis

Two trained qualitative analysts independently coded the transcripts from both Phase 1 (pre-use) and Phase 2 (during/after use), with discrepancies resolved through team discussion. Codes were first grouped into themes aligned to UTAUT domains and to our proposed extensions. We then used inductive coding to allow for the identification of any emergent themes, particularly those related to health-related topics and competing concerns.

Process data from brief weekly check-in calls were also reviewed for supporting evidence or context, with findings integrated into the broader thematic framework. Final themes were then mapped to the UTAUT model to understand their role in influencing the use of the app.

## Results

The majority of the sample were male (60%), white (87%), and had an income under $60,000 per year (87%), with a mean age of approximately 72 years (Table 1). Most participants reported good or fair health (93%). In addition to COPD, participants reported other health conditions such as Type 2 diabetes, emphysema, atrial fibrillation, kidney issues, asthma, sciatica, high blood pressure, prostate issues, and anxiety. Four reported using tobacco.

**Table 1:** Demographic and technology use characteristics.

Variable	*n* (%)
Total	15
Age (mean)	71.6
Sex	
Male	9 (60%)
Female	6 (40%)
Race/Ethnicity	
White	13 (87%)
African American	2 (13%)
Income	
$0–$24,999	3 (20%)
$25,000–$59,999	10 (67%)
$60,000–$99,999	1 (7%)
$100,000–$249,999	1 (7%)
Self-rated health	
Good	9 (60%)
Fair	5 (33%)
Poor	1 (7%)
Have a smartphone	
Yes	13 (87%)
Frequency of digital technology use	
Often	1 (7%)
Sometimes	3 (20%)
Rarely	6 (40%)
Never	5 (33%)
Comfort with technology	
Very comfortable	4 (26%)
Somewhat comfortable	10 (67%)
Somewhat uncomfortable	1 (7%)

Most (87%) reported owning a smartphone, but 73% indicated they rarely or ever used technology. All but one participant reported feeling somewhat to very comfortable with technology.

### Major themes

#### Prior health and technology perceptions

Throughout the Phase 1 interviews, participants cited both health-and technology-related barriers. Participants often struggled with changing health behaviors to become healthier, faced challenges such low motivation, lack of knowledge, or overwhelm from the steps required. Many participants said they struggled with remembering to take medications or keep medical appointments, while others said they did not find performing healthy behaviors enjoyable. Furthermore, many participants categorized their COPD as “mild,” so often prioritized attending to other health issues over attending to this health condition. Participants also characterized their past technology experiences as intimidating or challenging, with most citing difficulties regarding navigating apps and remembering passwords. Most expressed a lack of confidence in using technology, particularly if they did not have significant help setting up and learning the app from others.

### Main UTAUT constructs

#### Effort expectancy

In Phase 1 interviews, participants cited myriad smartphone barriers, including those related to effort expectancy and their anticipated difficulties using the app (Table 2). Although many participants initially struggled with navigation, they indicated in Phase 2 that they were able to learn how to use the app and access its features. By the end of the study, several had incorporated it into their daily routine.

**Table 2:** Comparison of UTAUT constructs in phase 1 with findings in phase 2.

Considerations reported prior to app introduction	Themes during 4-week trial period	Relevant quote
App effort expectancy		
Navigating different screens and tasks perceived as difficult	Although challenges were common during the initial introduction, barriers were overcome with repeated engagement	At first I thought oh crap, what the heck can I get myself into? I wasn't going to be able to do this Because, like I say, I can't navigate myself around a computer.- Participant 5
Learning new technology would be too much work to engage with frequently	Many participants found ways to integrate app engagement as part of their daily routine	Getting into the rhythm [with the app]…felt good…felt like a normal activity during the day- Participant 3
App performance expectancy		
App must be relevant to interests	Many participants found topics relevant to their interests, including weight loss, sleep, and mindfulness	Just being mindful on a daily basis (first thing in the morning) going through these paces, tracking my weight and blood pressure. It's positive because it keeps me aware of my physical condition. I can monitor, and if it's problematic, it's positive to go through paces just to be mindful and keep track of things.-Participant 1
App must provide useful information	Those with mild symptoms reported that the main COPD content was not relevant. Some who were less engaged with healthcare did not find use in the app overall	I'm sure that it would be helpful for somebody that has multiple things that they do to control their condition. But I really don't do anything. So I mean it didn't really help me control anything, ‘cause I don't really have anything to control. –Participant 7
App must show its utility in addressing health concerns	Participants discovered that the information could empower them to make healthcare changes, and appreciated health trackers and reminders to keep them on track	A lot of times if we don't know the whys or what to do [about our condition], you could feel helpless and overwhelmed. This way, I can access this information then maybe see “Hey, I got to make some drastic lifestyle changes.” As opposed to leaving in your doctor's hands…The more information you have at your disposal, going from the “why does this happen” to what to do about it, information is king, to allow you to have a hand in managing your condition.-Participant 1
Social influence		
Uncertainty about getting valuable information from an online source rather than from a doctor	Participants expressed frustration that they could not talk to a live coach, and had to communicate via text	[The coach] writes something and I write something back, OK, And it, it helps a little bit….OK, but it's not a video. It's not, it's not really a coach…And you can't express your feelings to words on a screen. We have to talk to an individual.-Participant 3
Doctors and family members as the main sources for influence to use the app	While participants still relied on loved ones for tech support, they found encouragement for continued use through app forums	It's nice to know that you know, like with my condition, that I'm not the only one, I'm part of a bigger community and I can see what works for others-Participant 4
Facilitating conditions		
Need technical support and training to become engaged in app	Participants often reached out to family members, friends, the IT support line, and study staff for help. However challenges with receiving assistance from IT support influenced perceptions of app use experience for many	They told me to leave a message, and they'd get back to me in 24 h. I didn't leave a message. I figured if it wasn't important to them, it wasn't important to me. -Participant 5
Technology self-efficacy		
Lack of confidence using technology	Many participants expressed nervousness at engaging with the app at first, but gained confidence over time	It went amazingly better than what I thought it would. … it's not that hard.- Participant 5

#### Performance expectancy

Other factors, such as app relevance to their health needs (performance expectancy), continued as key drivers of engagement and perceived utility throughout the study period. A common theme among participants, particularly those with milder illness, was the main app content about COPD was less relevant to their needs. Most participants appreciated the other information presented by the app (for example, weight loss or blood pressure management), and many said they learned something new about how to manage their health. Participants comments indicated they felt the app helped them better manage their health and learn specific health behaviors or techniques, and that their motivation for continuing was to address other current or emergent health issues rather than COPD. Two participants expressed continued low motivation; they were less likely to feel the app was relevant to their health goals and lack of desire to change their behavior.

#### Social influence

Participants indicated that the encouragement and companionship found in forums often inspired continued use. However, participants were wary to accept information from an online coach instead of their doctor, particularly since video chats were disabled for the study-based accounts. Many indicated their dissatisfaction with health advice from a text-based chat, and human connection for both health advice and technical support was still desired.

#### Facilitating conditions

Participants drew upon several sources of support when they encountered a challenge with the app. Identified challenges included tablet setup, account onboarding, or troubleshooting challenges that were specific to the study version of the app (e.g., videos not playing). Participants were quick to access assistance from the app helpline, study team, and sometimes family or friends. Almost all participants in the study indicated looking to some source of assistance over the course of the study.

Due to capacity limitations, however, technical support was often not able to assist with specific study account issues or questions. Many cited they felt like the support team didn't attend to their concerns and were often frustrated with what they perceived to be a gap in their support. This was perceived as a negative experience and like their needs were not important to the IT team.

### Technology self-efficacy

Although lack of self-efficacy was initially cited as a main barrier to technology use, particularly for more complex tasks such as meal tracking, confidence grew over time as participants became more familiar with the app. Approximately 2/3 of the sample expressed doubt when encountering the tablet and app, but confidence increased over the course of the study. However, one participant did not ever feel comfortable with the app, and felt that engagement remained difficult.

#### Competing concerns

In Phase 1, participants indicated that they were often overwhelmed with other life concerns that took priority over technology use. Stress from duties such as caregiving or keeping schedules organized were seen as deterrents to spending the time to learn and engage with technology. Similarly, in Phase 2, many participants cited competing concerns that disrupted their ability to engage with the app as frequently as they desired. These included their own health issues, particularly emergent issues such as COVID-19 or an injury, as well as needs of their family. Several participants indicated larger life issues, such as funerals and deaths in the family, impacted their ability to fully engage with the app. Other responsibilities, including babysitting grandchildren, could also be time consuming and reduce time available for app engagement.

#### Intention for continued use

Despite overall positive reactions to the app, interest in continuing use was mixed, often based on perceived relevance to their current COPD disease severity. One participant, who had a more severe case of COPD, felt the app was useful for managing her symptoms and said she would be likely to continue to use it. Some participants found other characteristics of the app helpful, such as the meal tracking, and cited an interest in continuing. However, participants who felt a lack of overall relevance of the app indicated they would not be likely to use it. Another key consideration for the level of willingness to continue to use an app such as this one was influenced by potential costs involved; many participants indicated they would not be able to afford costly up-front or monthly charges to engage with this content.

## Discussion

This study by a large health organization trialed use of a COPD smartphone app among older adults with limited prior health technology engagement. Participants were provided with a tablet pre-loaded with a smartphone app that addressed both COPD and other topics related to health and wellbeing and were interviewed about their experiences with the app, guided by the UTAUT. Despite initial hesitance on the part of many, participants were able to learn how to navigate the app and integrate it into their everyday lives. Comparisons of barriers noted prior to and after app introduction revealed that many effort expectancy barriers (e.g., hesitance to use technology, difficulty with app navigation) were overcome, while others, including facilitating conditions such as availability of technical support, persisted throughout. Once using the app, positive perceptions of performance expectancy were important facilitators for continued use throughout the study period. This process revealed several considerations for continued roll out of health-related apps.

App navigation was initially cited as an issue for many of these participants, with challenges including moving through different sections of the app, properly entering foods into calorie trackers, and adding tasks and habits. However, in almost all cases, these challenges had greatly reduced by the end of the study, with participants indicating that the app became easier to use over time as their familiarity increased.

This echoes past work that has illustrated that after a trial period, older adults can perceive technology as more approachable (23, 31). These instances can often serve to build confidence in technology use and demonstrate that devices and apps are not as difficult as they may initially appear. However, it is critical to note that all participants reported reaching out for help within the study, pointing to the continued need for ongoing support from family, friends, technical support, or other supports within the community to help gain this confidence and increase their ability to engage with technology.

Performance expectancy has been linked across several studies to the likelihood of technology adoption among older adults (26). Many participants indicated that their COPD was mild, and didn't feel that it required as much maintenance compared to other health-related concerns, leading to lower initial expectations of performance expectancy; however, many participants quickly found other areas of interest with app content It was common for participants to indicate that they spent most of their time exploring areas such as healthy eating, weight loss, stress relief, and sleep improvement to focus on their health in a broader sense. This points to the interrelatedness of health conditions that weave within the context of participants’ daily lives; apps that can provide an overall offering of health information may be perceived as more beneficial. Furthermore, apps that allow users to control the pace, priority of information, and desired amount of reinforcement or reputation may be useful to guide and support action. Participants' desire for an expanded set of topics available for articles and chat rooms underscores the importance of relevance for both initiation and continued use of a health app.

For many, healthcare organizations serve as critical entry points into the mHealth space, particularly for those with less digital experience. Recommendations from doctors or promotion by an insurance provider may increase awareness and use of applications that are tailored for specific health conditions. In addition to this recommendation, it is important to note that the continued desire for human connection was a central theme throughout. The lack of a synchronous video connection with a coach was seen as a limitation and that their concerns would not be fully understood through words alone. When recommending health apps for managing health conditions, providers should consider the range of needs and preferences for human-centric support that older adults may desire to accompany technology-based solutions.

The presence of competing life concerns was consistent theme across all interview time points. Several participants indicated that they had other, more pressing health issues, including uncontrolled high blood pressure and a hip that needed replacement. These issues often took precedence for participants, both in terms for information seeking and health behaviors. App engagement was also sometimes interrupted by life events, such as having a death in the family or having to care for loved ones. Several examples of these other life priorities arose within the study. This emphasizes the fact that no health condition occurs in a vacuum; other life stressors will always be present and may take precedence over the health condition that is specified in the app design.

It is vital to place the UTAUT within the larger life context and to consider potential moderators such as larger life events and caregiver responsibilities that may interfere with facilitating conditions to app use, or even disrupt the link between intention and app use. App designers and health systems alike should acknowledge that the app itself is not the primary focus for end users, who are grappling with a complex interplay of factors ranging from financial concerns to day-to-day tasks.

Apps that can address health from a whole-person care perspective (32) may have more value as users are able to access tools that integrate with their own personal goals and needs such as stress management, sleep improvement, or weight control. Recommending apps to the older adults with a sole focus on a certain disease or condition may represent a missed opportunity for patient engagement since health priorities may shift and evolve over time, especially for older adults with multiple comorbidities. However, given limited bandwidth, app function must allow for patients to easily navigate to clear, relevant information. This need for whole-person care can be illustrated by the variability of preferences and experiences within the sample; although the group of participants were selected based on narrow inclusion criteria, even seemingly similar individuals have specific preferences for use.

While this study was too brief to capture long-term behavior change, it reinforced that health information was highly valued by most participants, and many were eager to learn more about COPD and other health-related topics. Maximizing this interest through providing many different format options (e.g., article, video, health coach) can allow access to this content in a way that best meets patients' learning needs. Providing content in a format that is enjoyable to patients is also of value; many participants found the app entertaining and therefore integrated it into their daily routine. This routinization may help with allowing for the more consistent monitoring of disease symptoms over time and increasing adherence to medication, prescribed health behaviors, and doctors' appointments. Taken together, these findings echo past studies that characteristics of the technology (e.g., user experience, technical issues), health-based themes (e.g., the disease, health literacy, and care team role), and social and personal factors (e.g., societal and cultural aspects, demographic factors) all play a part in influencing uptake and continued use (8, 13, 33, 34).

### Limitations

One limitation of this study was the small sample size of 15 individuals who were all located within a specific geographical area, which limits generalizability of the study results. Future work should study these factors more in-depth using a larger, more diverse sample of older adults to ensure that other contextual factors, technology perceptions, and health and life circumstances are represented. Other models, such as the Technology Assessment Model (TAM), may provide opportunities for more exploration of emotion or affect in relation to technology compared to the UTAUT (35). However, the UTAUT's inclusion of factors such as social influence and facilitating conditions are able to capture a broader sense of the users' larger context for engagement. Future studies should consider integrating more of an emphasis of affect into the performance expectancy construct of the UTAUT to ensure these factors are assessed. Another limitation was that many upstream barriers to technology engagement were removed. For example, barriers such as lack of technology, awareness, or technical support were removed in this context. However, participants noted they had never heard of this app or similar offerings, suggesting that within a larger context, lack of awareness of potential app-based health solutions, coupled with hesitance due to unfamiliarity with technology and/or inability to afford the technology and internet connection needed for use, may present significant barriers to engagement.

## Conclusion

Guided by the UTAUT, this study illustrates the importance of a number of complex, interrelated factors that may impact uptake and use of a health app among low technology-engaged older adults. Features such as a focus on whole-person care should be emphasized so that users can curate a relevant, enjoyable app experience that can account for multiple comorbidities and life stressors. Family members and/or community support may also play a key role in providing early technical support by helping to get older adults set up with technology and demonstrating app use. More work is needed to understand the upstream factors that may inhibit use, with an emphasis on understanding how to offer robust social- and technology-based support to influence app uptake and continued engagement.

## Data Availability

The datasets presented in this article are not readily available because data are not available for public use. Requests to access the datasets should be directed to jon.black@highmarkhealth.org.
